# The Face Inversion Effect in Deep Convolutional Neural Networks

**DOI:** 10.3389/fncom.2022.854218

**Published:** 2022-05-09

**Authors:** Fang Tian, Hailun Xie, Yiying Song, Siyuan Hu, Jia Liu

**Affiliations:** ^1^State Key Laboratory of Cognitive Neuroscience and Learning and IDG/McGovern Institute for Brain Research, Beijing Normal University, Beijing, China; ^2^Beijing Key Laboratory of Applied Experimental Psychology, Faculty of Psychology, Beijing Normal University, Beijing, China; ^3^Department of Psychology & Tsinghua Laboratory of Brain and Intelligence, Tsinghua University, Beijing, China

**Keywords:** face inversion effect, deep convolutional neural network, VGG-Face, face system, AlexNet

## Abstract

The face inversion effect (FIE) is a behavioral marker of face-specific processing that the recognition of inverted faces is disproportionately disrupted than that of inverted non-face objects. One hypothesis is that while upright faces are represented by face-specific mechanism, inverted faces are processed as objects. However, evidence from neuroimaging studies is inconclusive, possibly because the face system, such as the fusiform face area, is interacted with the object system, and therefore the observation from the face system may indirectly reflect influences from the object system. Here we examined the FIE in an artificial face system, visual geometry group network-face (VGG-Face), a deep convolutional neural network (DCNN) specialized for identifying faces. In line with neuroimaging studies on humans, a stronger FIE was found in VGG-Face than that in DCNN pretrained for processing objects. Critically, further classification error analysis revealed that in VGG-Face, inverted faces were miscategorized as objects behaviorally, and the analysis on internal representations revealed that VGG-Face represented inverted faces in a similar fashion as objects. In short, our study supported the hypothesis that inverted faces are represented as objects in a pure face system.

## Introduction

Faces are an important type of visual stimulus in human social life and interaction, conveying a wealth of characteristic information (e.g., identity, age, and emotion) (Bahrick et al., 1975; O'Toole et al., 1998; Rhodes et al., 2011). Previous studies have found that humans processed faces differently from ordinary objects (e.g., Tanaka and Sengco, 1997). A classic manifestation of face specificity was the face inversion effect (FIE) (Yin, 1969; Valentine, 1988), in which humans are disproportionately less likely to recognize a face correctly when it is inverted than when an object (e.g., a cup) is inverted. However, the underlying mechanism of the FIE remains unclear.

Neuroimaging studies have been conducted to investigate how face-selective regions respond to upright and inverted faces. They found that the fusiform face area (FFA) is activated more highly when processing upright faces than inverted faces (Kanwisher et al., 1998; Yovel and Kanwisher, 2005; Epstein et al., 2006; Mazard et al., 2006). Further, the neural FIE observed in the FFA is positively correlated with behavioral FIE, suggesting that the FFA is likely the neural basis of the FIE (Yovel and Kanwisher, 2005; Zhu et al., 2011). In contrast, the activation of lateral occipital cortex (LOC), which is specialized for processing objects (Malach et al., 1995; Epstein, 2005), is greater during processing inverted faces than upright faces (Haxby et al., 1999; Yovel and Kanwisher, 2005). Taken together, the double dissociation of upright and inverted faces is considered as evidence that they are processed by the face system and object system, respectively. However, the findings are inconclusive; for example, the FFA is still responsive to inverted faces (Kanwisher et al., 1998; Yovel and Kanwisher, 2005) and the LOC is still responsive to upright faces (Haxby et al., 1999; Yovel and Kanwisher, 2005). It is possibly because the FFA is interacted with the LOC (Haxby et al., 1999; Yovel and Kanwisher, 2005; Epstein et al., 2006) and cannot completely rule out the influences from the object processing system.

Deep convolutional neural network (DCNN), which is inspired by biological visual systems, is used to simulate human vision recently (Kriegeskorte, 2015; Parkhi et al., 2015; Simonyan and Zisserman, 2015; Krizhevsky et al., 2017; Liu et al., 2020; Song et al., 2020; Huang et al., 2021; Tian et al., 2021; Zhou et al., 2021). Here we used a representative DCNN for face recognition, VGG-Face (Parkhi et al., 2015), which is pretrained to identify faces only. In recent years, various deep learning methods have been used in face recognition systems (Fuad et al., 2021). Among the various methods, DCNN is the most popular deep learning method for face recognition (Fuad et al., 2021). Further, visual geometry group network-face (VGG-Face) is one of the most commonly used CNN models for face recognition (e.g., Ghazi and Ekenel, 2016; Karahan et al., 2016; Grm et al., 2017) and has shown successful performance of face recognition under various conditions (Ghazi and Ekenel, 2016). Therefore, we selected VGG-Face in the present study as representative of face recognition models. VGG-Face provides an ideal model for human face system, completely insulated from the interference of the object system. Here we asked how the artificial face system, VGG-Face, represented inverted faces.

## Methods

### Deep Convolutional Neural Networks

As a pure face system, VGG-Face (available in https://www.robots.ox.ac.uk/~vgg/software/vgg_face/) is pretrained with the VGG Face Dataset. The architecture of VGG-Face includes 13 convolutional layers and 3 fully connected layers (i.e., FC1, FC2, and FC3), and the FC3 is a 2,622-dimensional classifier, corresponding to the 2,622 face identities to be identified during pretraining (Parkhi et al., 2015).

To compare the FIE between face system and object system, we used AlexNet (available in https://pytorch.org/) as an object system, which was pretrained for classifying objects with the ImageNet data set (Krizhevsky et al., 2017). AlexNet has an eight-layer architecture; the first five layers are the convolutional layers, and the last three layers are fully connected layers (i.e., FC1, FC2, and FC3); the FC3 layer is a classifier of 1,000 units.

To examine the effects of network architecture and pretraining task on the FIE, we also used VGG-16 (Simonyan and Zisserman, 2015), which has the same network architecture as VGG-Face but the same pretraining experience as AlexNet. In addition, we used an AlexNet with the same pretraining experience of face recognition as VGG-Face using the VGG Face Dataset (Grm et al., 2017).

### Experiment Settings

#### The Face and Object Data Sets

**Figure 1:**
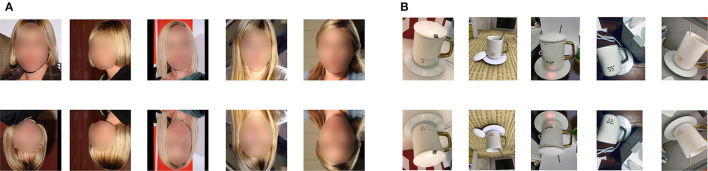
Example stimuli in our study. **(A)** Top, upright faces; bottom, inverted faces. **(B)** Top, upright objects; bottom, inverted objects.

We used a data set of 60 groups of images, of which 30 groups were face images and 30 groups were object images (Figure 1). Each group of face images contained images of one individual in different scenes, all selecting from CASIA-WebFace database (Yi et al., 2014). To rule out the effect of the pretrained face identities on the transfer training, the face identities in our data set did not overlap with those in VGG-Face pretraining data set. Each group of object images contained images of one specific cup in different scenes, and all object images were selected from the Internet. All the images were evaluated to ensure that the face or object in each image was complete. In each group, there were 75 images for transfer training, 25 images for validation, and 50 upright images and 50 inverted images for testing. The inverted stimuli were obtained by rotating the upright images 180 degrees. Thus, a total of 12,000 images were used in this study, with 4,500 images used for training, 1,500 images used for validation, and 6,000 images used for testing (1,500 upright faces, 1,500 inverted faces, 1,500 upright objects, and 1,500 inverted objects). Before training, the input images were normalized to a uniform size of 224 × 224 and normalized according to the mean and standard deviation of the ImageNet database (mean = [0.481,0.457,0.398], std = [0.237, 0.232, 0.231]).

#### Transfer Learning

The transfer learning included a training period and a validation period. During the training period, the DCNNs were trained to classify images of 30 face identities and 30 cup identities. All network parameters of the pretrained DCNNs were frozen except for the last FC3 layer, using DNNBrain (Chen et al., 2020) based on the PyTorch. The FC3 layer of the DCNNs was changed to an FC classifier of 60 units to fit the training task. The 60-group data set was presented to DCNNs, and the classifiers of the FC3 layers were trained. A total of 100 epochs were performed in the training period, and the loss value of the network was generated after each epoch. The loss fluctuates within a stable range when the training is finished. After the training period, other exemplars of the 60 faces and cup identities were presented to the DCNNs in the validation period, and the recognition accuracy of validation was evaluated.

#### Testing Experiment

After transfer learning, we presented upright faces, inverted faces, upright objects, and inverted objects to the DCNNs in the testing experiment. The recognition accuracy was obtained by comparing the output and input identities of each image. Further, for classification error analysis, we examined the errors the DCNNs made in different conditions (i.e., whether upright/inverted faces were classified as objects and whether upright/inverted objects were classified as faces).

To further explore how the DCNNs represented upright and inverted images, we used representational similarity (RS) analysis (Kriegeskorte et al., 2008) to examine the RS of different stimulus identities in the DCNNs. We used DNNBrain (Chen et al., 2020) to extract the activation values of the 6,000 testing images in the three FC layers of the DCNNs. For both networks, the activation values of 4,096 units in FC1, 4,096 units in FC2, and 60 units in FC3 were extracted. The activation values of the 50 upright images and 50 inverted images of each identity were averaged, respectively, and for each identity, the activation patterns of upright and inverted conditions were obtained in each FC layer. Then, Pearson's correlation was calculated to obtain the representation similarity of different stimulus identities in each FC layer.

## Results

### Transfer Learning of VGG-Face

VGG-Face was trained to classify images of 30 face identities and 30 cup identities in transfer learning. The training performance reached stability after 50 epochs. The validation accuracy of VGG-Face was 67.8%, significantly higher than the random level (random accuracy = 1.67%), which indicated that the transfer learning of VGG-Face was successful.

### FIE in VGG-Face

**Figure 2:**
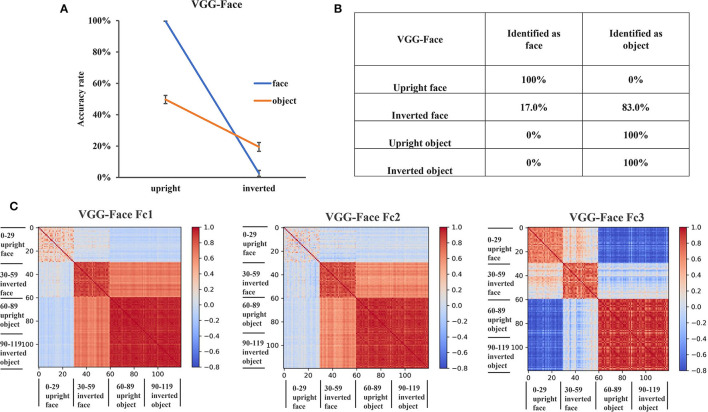
Recognition performance and representations of VGG-Face. **(A)** The recognition accuracy of the upright and inverted faces and objects of VGG-Face. The error bars denote the standard error of the mean across the 30 groups of images in each condition. **(B)** The classification confusion matrix of VGG-Face. The percentage in the matrix denotes the classification errors of VGG-Face in each condition. **(C)** The representational similarity matrix of the three FC layers in VGG-Face. The color in the matrix indicates correlation values between activation patterns of different stimulus identities, with cool color in the matrix indicating low correlation and the warm color indicating high correlation.

We first examined whether there was an FIE in VGG-Face as a pure face system. We performed a two-way ANOVA analysis on recognition accuracy with orientation (upright, inverted) and stimuli category (faces, objects) as factors (Figure 2A). The main effects of both orientation [*F*_(1, 116)_ = 824.76, *p* < 0.001] and stimulus category [*F*_(1, 116)_ = 55.90, *p* < 0.001] were significant. There was an interaction between stimulus category and orientation [*F*_(1, 116)_ = 228.58, *p* < 0.001]. The accuracy of inverted images decreased more in face condition [*F*_(1, 116)_ = 960.8, *p* < 0.001] than in object condition [*F*_(1, 116)_ = 92.47, *p* < 0.001], indicating that there was an FIE in VGG-Face.

To further investigate why the VGG-Face showed an FIE, we examined the classification errors of VGG-Face in different conditions. While all upright faces were classified as faces and all upright and inverted objects were classified as objects, only 17% of the inverted faces were classified as faces and 83% were classified as objects in VGG-Face (Figure 2B). This result suggested that VGG-Face showed an FIE because it tended to classify inverted faces as objects.

### VGG-Face Represented Inverted Faces Similarly as Objects

The misclassification of inverted faces as objects behaviorally suggested that inverted faces might be represented more similarly to objects in VGG-Face. To test this intuition, we performed the RS analysis in the three FC layers of VGG-Face. We found that in FC1 and FC2, the representation of inverted faces was clustered with that of the objects, rather than with that of upright faces (Figure 2C). In FC1 layer, the RS within upright faces (0.27) was much lower than that within inverted faces (0.84) and within objects (0.94). Importantly, the RS between inverted faces and objects was 0.66, while the RS between inverted and upright faces was only −0.037, and that between upright faces and objects was −0.07. The results in FC2 layer showed a similar pattern as in FC1. That is, the RS within upright faces (0.12) was much lower than that within inverted faces (0.82) and within objects (0.91). The RS between inverted faces and objects was 0.59, while the RS between inverted and upright faces was only −0.002, and that between upright faces and objects was −0.013. In FC3 layer, we observed that the representations of upright faces, inverted faces, and objects were clustered into three clusters (Figure 2C). The RS within upright faces was 0.66, the RS within inverted faces was 0.71, and the RS within objects was 0.87. The RS between upright faces and inverted faces was 0.21, the RS between upright faces and objects was −0.68, and the RS between inverted faces and objects was −0.05. These results indicated that inverted faces were represented more similarly as objects than as upright faces in the FC layers of VGG-Face, providing representational basis for the behavioral results that VGG-Face tended to classify inverted faces as objects.

### AlexNet Did Not Show an FIE

Having shown the FIE and revealed its internal representations in a pure face system, VGG-Face, we next examined whether the FIE was specific to the pure face system or would also be observed in an object system. Here we used AlexNet (Krizhevsky et al., 2017), which was pretrained for object categorization with ImageNet. The same procedure of transfer learning was applied for AlexNet as for VGG-Face, the training performance reached stability after 40 epochs. After training, the validation accuracy of AlexNet was 69%.

**Figure 3:**
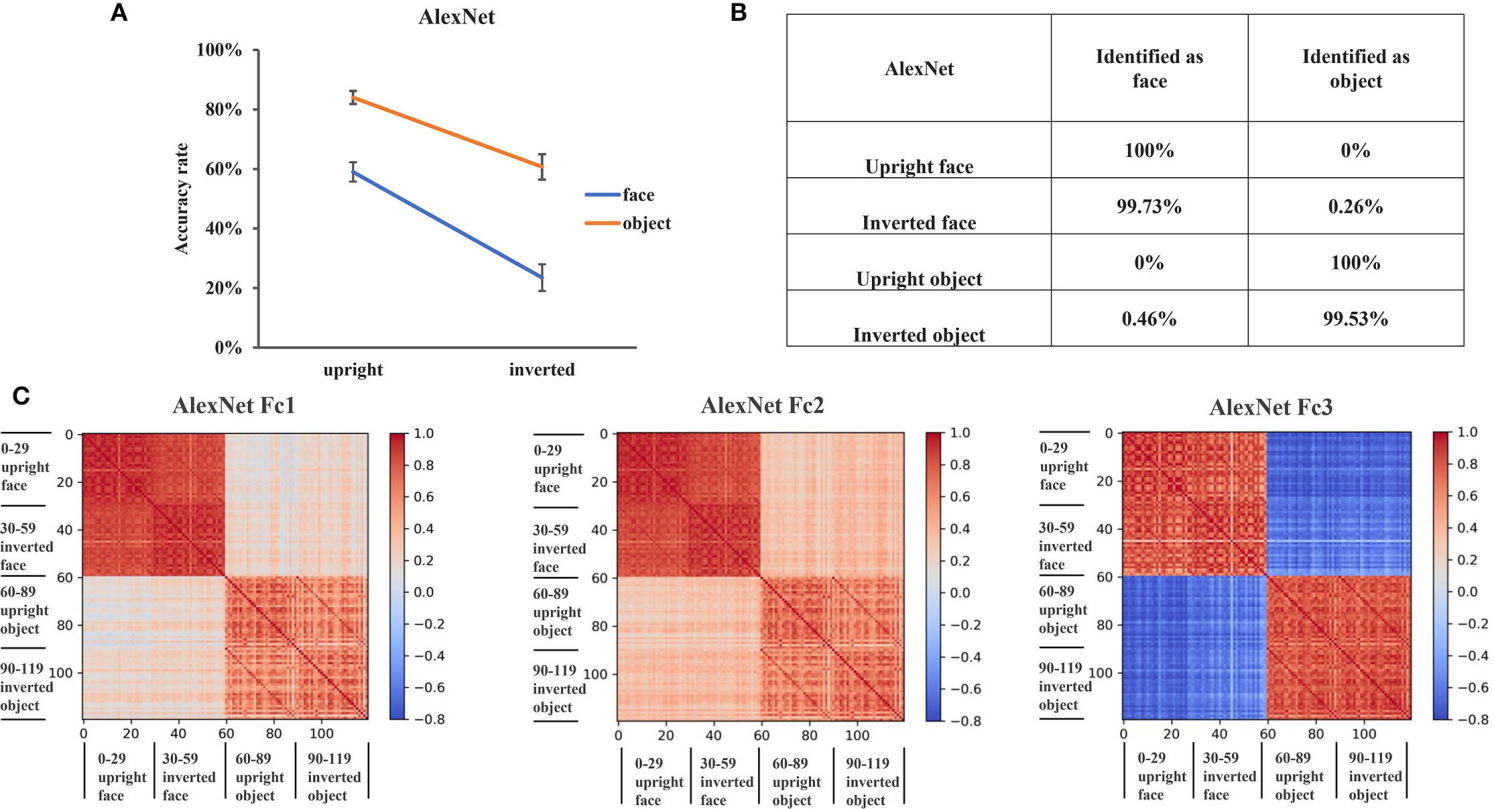
Recognition performance and representations of AlexNet. **(A)** The recognition accuracy of the upright and inverted faces and objects of AlexNet. The error bars denote the standard error of the mean across the 30 groups of images in each condition. **(B)** The classification confusion matrix of AlexNet. The percentage in the matrix denotes the classification errors of AlexNet in each condition. **(C)** The representational similarity matrix of the three FC layers in AlexNet. The color in the matrix indicates correlation values between activation patterns of different stimulus identities, with cool color in the matrix indicating low correlation and the warm color indicating high correlation.

To examine whether there was an FIE in AlexNet, we performed a two-way ANOVA of orientation (upright, inverted) by stimuli category (faces, objects) on recognition accuracy. The main effects of both orientation [*F*_(1, 116)_ = 62.27, *p* < 0.001] and stimulus category [*F*_(1, 116)_ = 70.43, *p* < 0.001] were significant, but the interaction between stimulus category and orientation was not significant [*F*_(1, 116)_ = 2.57, *p* = 0.11] (Figure 3A). This result indicated that there was no FIE in AlexNet. We also performed a three-way ANOVA of orientation (upright, inverted), stimuli category (faces, objects), and network (AlexNet, VGG-Face), and the significant three-way interaction [*F*_(1, 232)_ = 40.41, *p* < 0.001] indicated that VGG-Face showed a greater FIE than AlexNet.

Then, we examined the classification errors of AlexNet in different conditions. In contrast to VGG-Face where most inverted faces were classified as objects, 99.7% of the inverted faces were classified as faces in AlexNet (Figure 3B). Besides, all upright faces were classified as faces, and all upright objects and 99.5% inverted objects were classified as objects in AlexNet. These results suggested that inverted faces were represented similarly as upright faces, rather than objects, in AlexNet.

To test this hypothesis, we performed the RS analysis in the three FC layers of AlexNet. We found that the representations of faces and objects were grouped into two clusters in AlexNet, regardless of the upright and inverted orientations (Figure 3C). In all FC layers, the within-category RS was greater than the between-category RS. That is, the RS between upright and inverted faces (FC1 layer was 0.86; FC2 layer was 0.86; FC3 layer was 0.78) and the RS between upright and inverted objects (FC1 layer was 0.62; FC2 layer was 0.64; FC3 layer was 0.79) were greater than the RS between faces and objects (FC1 layer was 0.19; FC2 layer was 0.34; FC3 layer was −0.63). These results indicated that upright and inverted faces were similarly represented in AlexNet.

### VGG-16 Pretrained With Object Classification and AlexNet Pretrained With Face Recognition

The different FIEs observed in VGG-Face and AlexNet might be accounted for either by their different network architectures or by different pretraining tasks (face recognition vs. object classification). In order to explore the effects of pretraining experience and network architecture on FIE, two more experiments were conducted. First, we used VGG-16 (Simonyan and Zisserman, 2015), which has the same network architecture as VGG-Face but the same pretraining task of object classification as AlexNet. Second, we used an AlexNet trained from scratch with the same pretraining experience of face recognition as VGG-Face (Grm et al., 2017).

The same procedure of transfer learning was applied, and the training performance reached stability after 50 epochs. After training, the validation accuracy was 65.4% for VGG-16 and 64.7% for AlexNet.

**Figure 4:**
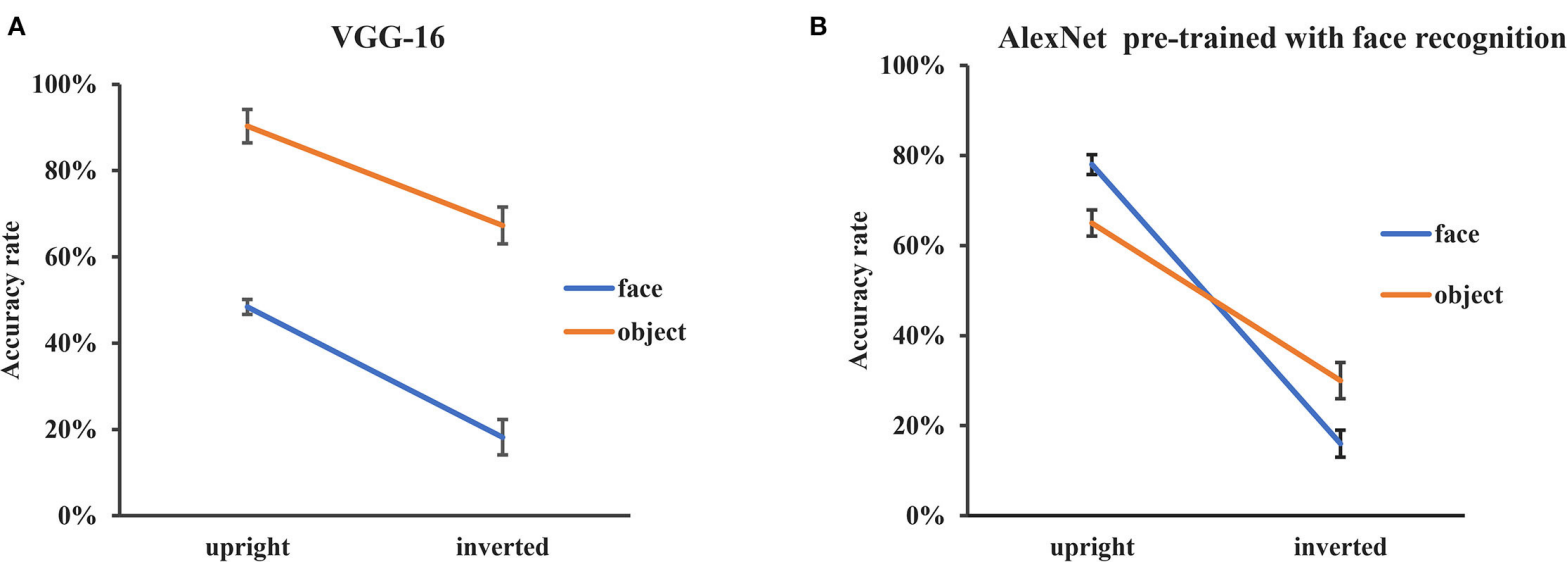
Recognition performance of VGG-16 and AlexNet pre-trained with face recognition. **(A)** The recognition accuracy of the upright and inverted faces and objects of VGG-16 pretrained with object classification. **(B)** The recognition accuracy of the upright and inverted faces and objects of AlexNet pretrained with face recognition. The error bars denote the standard error of the mean across the 30 groups of images in each condition.

For VGG-16, we performed a two-way ANOVA of orientation (upright, inverted) by stimuli category (faces, objects) on recognition accuracy. The main effects of both orientation [*F*_(1, 116)_ = 51.73, *p* < 0.001] and stimulus category [*F*_(1, 116)_ = 150.98, *p* < 0.001] were significant, but the interaction between stimulus category and orientation was not significant [*F*_(1, 116)_ = 0.96, *p* = 0.32] (Figure 4A). That is, the VGG-16 pretrained with object classification did not show an FIE.

Similar analysis was performed for the AlexNet pretrained with face recognition. The main effect of orientation [*F*_(1, 116)_ = 223.96, *p* < 0.001] was significant and the main effect of stimulus category was not significant [*F*_(1, 116)_ = 0.002, *p* = 0.96]. Importantly, there was an interaction between stimulus category and orientation [*F*_(1, 116)_ = 17.29, *p* < 0.001] (Figure 4B). The accuracy of inverted images decreased more in face condition [*F*_(1, 116)_ = 182.85, *p* < 0.001] than in object condition [*F*_(1, 116)_ = 58.37, *p* < 0.001], indicating that there was an FIE in the AlexNet pretrained for face recognition.

Taken together, the two network architectures showed similar FIE after pretrained with face recognition task, but showed no FIE after pretrained with object classification task. These results suggested that the observed FIE in DCNNs may result from pretraining experience of face recognition, rather than particular DCNN network architectures.

## Discussion

In this study, we used VGG-Face to examine whether there was an FIE in an artificial pure face system and how upright and inverted faces were represented in this system. We found that there was an FIE in VGG-Face and the FIE was stronger than that in AlexNet which was pretrained for processing objects. Further classification error analysis revealed that in VGG-Face, inverted faces were misclassified as objects behaviorally, and the analysis on internal representations revealed that the VGG-Face represented inverted faces in a similar fashion as objects. These findings supported the hypothesis that inverted faces are represented as objects in the face system. Although fMRI studies have revealed some neural basis of FIE, especially in the FFA (Yovel and Kanwisher, 2005), the results are inconclusive, which may be due to the fact that the face system is not completely insulated from object system in human brain. By using an artificial pure face system as well as a pure object system, our study provides a clearer account for the representations underlying FIE.

The FIE found in VGG-Face as a pure face system is consistent with previous human fMRI findings showing an FIE (i.e., higher response to upright than inverted faces) in the face-selective FFA, and the FIE in the FFA correlates with the behavioral FIE (Yovel and Kanwisher, 2005). Further, fMRI adaptation results provide a possible neural basis for the behavioral FIE by showing that the FFA was more sensitive to identity differences between upright faces than inverted faces (Yovel and Kanwisher, 2005). This finding fits nicely with our results that the RS within upright faces was much lower than that within inverted faces and objects in VGG-Face, indicating that different identities were more uniquely represented in upright faces than inverted faces. Our results are also consistent with a previous study which showed a similar FIE using pretrained VGG-Face (Elmahmudi and Ugail, 2019).

More importantly, we extended previous finding by revealing representations underlying the observed FIE. First, we found that VGG-Face misclassified the inverted faces as objects behaviorally. This result is in line with neuropsychological finding that a patient with object recognition impairment was severely impaired in recognition of inverted faces, but normal at recognition of upright faces (Moscovitch et al., 1997). Moreover, RS analysis showed that in VGG-Face, inverted faces were represented similarly as objects, while representation of upright faces was separate from those of inverted faces and objects. Together, these results provide novel and clear evidence for an account of human FIE that inverted faces are represented by general object mechanisms whereas upright faces are represented by mechanisms specialized for faces (Yin, 1969; Pitcher et al., 2011).

In contrast, the AlexNet and VGG-16 pretrained for object categorization did not show an FIE, and upright and inverted faces were similarly represented in AlexNet. This result is consistent with human fMRI results that the object-selective LOC shows similar sensitivity to face identities for upright and inverted faces (Yovel and Kanwisher, 2005). Notably, although the AlexNet did not show a behavioral FIE in our study, it is reported that responses of face-selective units in untrained AlexNet responded more highly to upright faces than inverted faces (Baek et al., 2021). The discrepancy may be caused by different analysis levels (behavioral level vs. single unit response level) or different layers (FC layers vs. convolution layers). It will be interesting to examine whether untrained AlexNet will show an FIE behaviorally.

In sum, the present study showed an FIE in an artificial pure face system. Our study highlighted the important role of pretraining of face identification for a system to show the FIE; future studies are awaited to examine whether other DCNN networks or other types of deep learning models pretrained with face identification tend to show a similar FIE and whether the exposure of face stimuli or the task of face identification is more critical. Additionally, our study provided evidence for a possible mechanism of the FIE that inverted faces are represented as objects while the upright faces are represented differently from objects and inverted faces. Human behavioral studies have suggested that processing of upright faces is special in that they are processed in a holistic manner, while processing of inverted faces and non-face objects is based on featural information (Young et al., 1987; Tanaka, 1993; Farah et al., 1995; Tanaka and Sengco, 1997; Maurer et al., 2002; Tanaka and Farah, 2006). Future studies are invited to examine in what manners upright and inverted faces are represented in artificial face system. Finally, our study may inspire more researchers to use DCNNs to explore the cognitive mechanisms of face recognition, especially the problems that cannot be solved with human subjects because of some limitations (such as ethics, experience, and workload).

## Data Availability Statement

All codes for analyses and examples of datasets are available on https://github.com/Helen-Xie-Nep/DCNN-Face-inversion.

## Author Contributions

JL and SH designed the research. HX collected the data. HX and FT analyzed the data. FT, JL, and YS wrote the manuscript. All authors contributed to the article and approved the submitted version.

## Funding

This work was supported by the National Natural Science Foundation of China (31861143039, 31872786).

## Conflict of Interest

The authors declare that the research was conducted in the absence of any commercial or financial relationships that could be construed as a potential conflict of interest.

## Publisher's Note

All claims expressed in this article are solely those of the authors and do not necessarily represent those of their affiliated organizations, or those of the publisher, the editors and the reviewers. Any product that may be evaluated in this article, or claim that may be made by its manufacturer, is not guaranteed or endorsed by the publisher.
